# Second breast cancers in a Tuscan case series: characteristics, prognosis, and predictors of survival

**DOI:** 10.1038/sj.bjc.6604481

**Published:** 2008-07-15

**Authors:** S Ciatto, N Houssami, F Martinelli, R Bonardi, F H Cafferty, S W Duffy

**Affiliations:** 1 Centro per lo Studio e la Prevenzione Oncologica (CSPO), Istituto Scientifico della Regione Toscana, Florence, Italy; 2 Screening and Test Evaluation Program (STEP), School of Public Health, University of Sydney, Sydney, Australia; 3 Department of Mathematics and Statistics, Centre for Epidemiology, Cancer Research UK, Wolfson Institute of Preventive Medicine, London, UK

**Keywords:** breast cancer, contralateral cancer, disease-specific survival, ipsilateral breast relapse, prognosis

## Abstract

Little is known about long-term outcomes following a second breast cancer diagnosis. We describe the epidemiology, characteristics and prognosis of second breast cancers in an Italian cohort. We identified women with two breast cancer diagnoses from 24 278 histology records at a Tuscan breast cancer service between 1980 and 2005, and determined their survival status. Disease-specific survival from second diagnosis was examined using Cox regression analyses. Second cancers were identified in 1044 women with a median age of 60 years. In all 455 were ipsilateral relapses and 589 were contralateral cancers. Median time between first and second diagnosis was 63.4 months. The majority of second cancers was small invasive or *in situ* tumours. Estimated 10-year survival from a second cancer diagnosis was 78%. Survival was poorest when the second cancer was large (HR=2.26) or node-positive (HR=3.43), when the time between the two diagnoses was <5 years (HR=1.45), or when the diagnosis was in an earlier epoch (HR=2.20). Second tumours were more likely to be large or node-positive if the first breast cancer had these features. Prognosis following a second breast cancer in this cohort was generally good. However, large or node-positive second tumours, and shorter intervals between diagnoses were indicators of poorer survival.

Breast cancer (BC) is estimated to be the most prevalent cancer in the world (Parkin *et al*, 2005). Earlier diagnosis and better therapy have improved survival from BC. Information relevant to the care of this group of such women, who have an elevated risk of a second BC, is relevant to population health and clinical practice. Few studies have examined a second BC in well-defined series, and little is known about the characteristics and long term outcome in affected women (Grunfeld *et al*, 2002).

We report on the largest clinically defined cohort (to date) of second BC events, based on consecutive cases in a major breast service in Tuscany, and including women with ipsilateral breast relapse (IBR) and contralateral breast cancer (CBC). We aim to describe the epidemiology and clinical characteristics of second BCs, and to identify factors associated with poorer survival following a second BC diagnosis. The focus of our study is therefore prognosis following second BC events and not predictors of its occurrence.

## Materials and methods

Eligible participants consisted of all women with a prior primary BC (invasive or *in situ*) who were diagnosed with a second metachronous BC (⩾6 months after the first cancer) in 1980–2005 at the study centre. We searched the clinical and pathology archives of the Centro per lo Studio e la Prevenzione Oncologica (CSPO), identifying women with histology records (surgical or needle histology) indicating two BC diagnoses separated by at least 6 months. Medical records were then reviewed to verify eligibility. Centro per lo Studio e la Prevenzione Oncologica is Florence's main breast screening and diagnostic service, and is the only centre in the region that provides follow-up services for women with BC. Surveillance consists of two-view mammography complemented by clinical examination (with more frequent clinical examination in the initial 5 years in women treated with breast conservation). Centro per lo Studio e la Prevenzione Oncologica archives have ongoing data linkage to population cancer and mortality registries in the Tuscan region.

We use the term second BC (‘second cancer’) when describing all cases, but define two subgroups eligible for inclusion: women with (1) ipsilateral breast relapse (IBR), who developed a second cancer in the breast that was previously affected (including those with breast relapse and concomitant axillary disease); and (2) women with CBC, who developed a second cancer in the opposite breast. Women presenting with metastatic cancer at diagnosis of the second BC, who represent a very small proportion of women, were not considered for this study as histology verification is inconsistently available in these cases; inclusion might bias estimates of survival from second cancer diagnosis; and owing to difficulty in ascertaining whether the metastases were related to the first or second cancer event. Women presenting primarily with nodal metastases or chest wall recurrences (following mastectomy for the first cancer) were not eligible for inclusion in this study.

Data retrieved from clinical records included date of birth; date of diagnosis, histology and pathological T and N category of the first BC; date of diagnosis, side, histology and pathological T and N category of the second cancer; presence or absence of symptoms at diagnosis of second cancer; surgical management; and date of last follow-up. Survival status was assessed directly for patients regularly followed up at CSPO or according to the regional Mortality Registry for cases lost to active follow-up. Linkage with the Mortality Registry for cause of death was complete to 31 December 2005, and ascertainment of outcomes was 97% complete to 31 December 2006. Data on treatment were not available for this study, but in our setting, it comprises radiotherapy following breast conservation and (since about 1988) adjuvant systemic therapy.

### Statistical analysis

Analyses are presented for all second cancers collectively and for each group (IBR and CBC). Descriptive data are reported on key variables including histology and tumour stage distribution for both the first and the second cancers. Time between occurrence of the first cancer and histological diagnosis of a second cancer (‘disease-free interval’, DFI), and time from second diagnosis to BC death, were calculated. When comparing features of the second tumour with features of the first tumour, data are paired and the McNemar's *χ*^2^ test was used. When comparing the IBR cases with the CBC cases, groups are independent and the *χ*^2^ test was used.

To determine the prognostic effect of features of the second cancer, we examined disease-specific survival from the second cancer diagnosis. Survival from diagnosis of the first cancer will be the subject of a separate study. For subjects who were alive at the end of follow-up or had died from a different cause, observations were censored at date of their last observation (normally 31 December 2006) or date of death. Ten-year survival was estimated using the Kaplan–Meier method. Multivariate Cox regression analysis was performed using the following covariates: (a) age at second cancer diagnosis (<50, 50–69, ⩾70 years); (b) time from first to second cancer (DFI); (c) stage (pT and pN category) of the second cancer; (d) time period in which the second cancer was diagnosed (1980–97, 1998–2005); and (e) type of second cancer (IBR or CBC). Models were fitted separately for IBR and CBC cases, as well as for all second cancers. Results are expressed in terms of hazard ratios (HR) and 95% confidence intervals (CI).

The Cox regression model was used to identify features of the second tumour that had the most significant adverse influence on prognosis following its diagnosis. Multivariate logistic regression analyses were then used to identify which features of the first BC predicted such second BC. The covariates under consideration were (a) age at first cancer diagnosis (<50, 50–69, ⩾70 years); (b) stage of the first cancer (pT and pN category); and (c) histology of the first cancer (invasive ductal, DCIS, other invasive). Note that this analysis is designed to predict particular features of a second tumour and not the occurrence of a second tumour.

All statistical analyses were performed using the STATA software, release 8.0.

## Results

Second BCs were identified in 1044 women from 24 278 breast histology records: IBR in 455 subjects and CBC in 589 (Table 1). The median age at diagnosis of the second cancer was 60 years (IQR 51–70). Follow-up was available in all but 36 cases (20 IBR, 16 CBC), which were excluded from outcome analyses but retained in descriptive analyses. The median follow-up period from the occurrence of the first cancer was 13.7 (IQR 9.0–18.1) years. There were 181 BC deaths and 62 from other causes (Table 1). Second cancers were more likely to be detected asymptomatically (67.0%) than symptomatically (33.0%, *P*<0.001). However, the proportion of symptomatic cases did not differ between IBR and CBC (*P*=0.21, Table 1).

The majority of second cancers was small invasive or *in situ* (77% were pTis or pT1). DCI comprised 12%, invasive ductal 56% and other histological types 32%. Of CBC cases, 56% were node negative, 20% were node positive, and in 24% node status was unknown. There was a significant difference between IBR and CBC with respect to the histological type of the tumour (*P*<0.0001), more IBR cases being DCIS, whereas more CBC cases were invasive lobular. Similarly, there was a significant difference between the two groups in the histological types of the first tumour (*P*<0.0001); more IBR had been diagnosed with DCIS previously compared with those with CBC (17.1 *vs* 5.8%). Significant differences in the distribution of pT categories between IBR and CBC were also seen for both first and second tumours (Table 1).

Table 2 summarises data on size (pT) of second tumours according to the size of the first tumour and the type of second cancer (IBR or CBC). For women diagnosed with CBC, the second tumour tended to be smaller than the first. Of those with known tumour size of both first and second cancers, 80.5% (409/508) of second cancers were pT1 or smaller, compared with 58.5% (297/508) of first cancers (*P*<0.0001). However, this difference was not observed in women with IBR, whose second tumour was more likely to be of a similar size to the first tumour (83.3% (334/401) of second cancers and 81.5% (327/401) of first cancers were pT1 or smaller, *P*=0.5).

**Figure 1:**
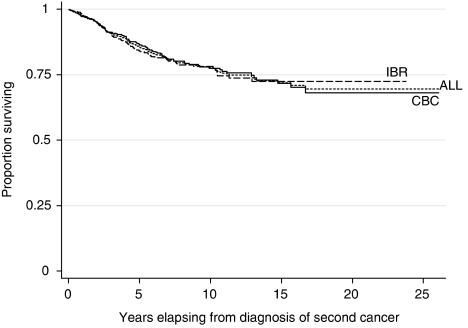
Kaplan–Meier disease-specific survival curves for ipsilateral breast relapse (IBR), contralateral breast cancer (CBC) and all second cancers.

Figure 1 shows Kaplan–Meier survival curves for IBR, CBC and all second cancers. The estimated 10-year survival rate from IBR was 77.9%, from CBC, 77.3% and overall in second cancers 77.6%. In univariate analysis significantly poorer survival was observed with younger (<50 years) and older (>69 years) age at second cancer diagnosis (*P*=0.03), a short interval from first to second cancer (*P*=0.001), larger tumour size (*P*<0.0001), positive nodes (*P*<0.0001), and cancers diagnosed during the earlier time period (*P*<0.0001). These effects were similar for IBR and CBC cases and so both groups were included in the final multivariate model. The multivariate Cox regression model is presented in Table 3 showing the proportional hazard ratios (HR) and 95% CI for second BC variables. The strongest predictor variables for survival in this model were pT and pN categories, although time between first and second cancer, and epoch of diagnosis of second cancer, also remained significant. There was no evidence of a difference in survival between IBR and CBC.

The Cox model indicates that second cancers that were 2 cm or larger or node positive had a poor prognosis. Table 4 summarises multivariate analysis of the features of the first cancer that predicted these features in the second tumour: women whose first cancer was large or node positive at diagnosis were more likely to have large or node-positive second cancer.

## Discussion

The characteristics of second BCs in women presenting to a major centre in Tuscany represents the largest series to date to include both IBR and CBC, with data on outcomes in almost all subjects. As BC survival is improving, clinicians will be providing care to increasing number of women at risk of developing a second cancer in either breast. Women who experienced IBR or CBC in this study were on average around 60 years of age when they experienced the second BC event. This is not an old population in the context of life expectancy in developed countries. These cancers are more likely to be diagnosed in asymptomatic women, and are generally smaller tumours than the initial cancer in the same women.

Overall 10-year survival from diagnosis of the second cancer was 77.6%, and the median time between first and second cancer diagnosis was 63.4 months. A shorter time from first till second cancer diagnosis was associated with poorer survival. Ten-year survival estimates in our study are similar to survival estimates of 75% observed for first invasive cancers in the Swedish two-county study (Tabar *et al*, 1992), and the 73% observed in first invasive cancers diagnosed in the 1990s in the West Midlands, UK (G Lawrence, personal communication).

Many studies have looked at IBR and reported on factors predicting local recurrence. We have taken a different approach by focusing on the identification of features associated with prognosis once a second BC has occurred (rather than predictors of recurrence) and have included CBC as well as IBR. Most studies of CBC have included small number of cases and limited follow-up (Grunfeld *et al*, 2002). The exception to this is a study that reported on stage-related survival in CBCs only and showed good prognosis in early-stage tumours (Schootman *et al*, 2006). We recognise that IBR and CBC are essentially different events and for this reason we have reported data separately in the two groups. Our summary of key features (Table 1) highlights both similarities and differences in second cancers. Variables associated with prognosis in survival analysis were similar for both IBR and CBC. We were unable to report on tumour grade and hormone receptor status because of incomplete or non-availability of such data. It should be noted that Rack and colleagues, in a study of locoregional breast relapse found that tumour grade was not independently associated with risk of death following relapse (Rack *et al*, 2003).

The distribution of times between first and second cancer diagnosis in this cohort (both IBR and CBC) provides evidence supporting long-term follow-up of women after BC. Currently, some groups recommend only limited follow-up (National Institute for Clinical Excellence, 2002; The Association of Breast Surgery @ BASO, 2005). However, our study suggests that a recommended follow-up of 10 years or even longer may be more appropriate (Smith *et al*, 1999; Khatcheressian *et al*, 2006). A recent study from Edinburgh, based on 108 relapses (including 35 cases of CBC) makes a similar point, and reports that treatable breast relapse occurred at a constant rate for at least 10 years (Montgomery *et al*, 2007). The benefits of extending surveillance must be weighed against potential disadvantages in terms of overdiagnosis and overtreatment although these effects are likely to be small for this cohort.

Surgical management of CBC was more often breast conservation than mastectomy, in keeping with the preponderance of small cancers in this group. In addition, most women with CBC had node-negative disease, although data on nodal status were not known in about a quarter of cases (nodes either not excised or data not notified to the study centre). Although local excision of in-breast relapse is associated with lower local control than salvage mastectomy (van der Sangen *et al*, 2006), a 40% breast conservation rate in women with IBR in our study might reflect feasibility of local excision as 62% were small tumours.

Our data do not represent complete population data; however, the study centre is the region's main breast diagnostic service with linkage to population registries, and is the only service in the Tuscan region which provides organised surveillance for women with a past BC history. We therefore consider that the majority of women in our case definition will have been identified and included. It is also relevant that our study concerned subjects with two BCs and does not allow calculation of predictors of the occurrence of a second cancer event nor rates of IBR and CBC. These were not within the scope of this evaluation; such data on these issues have been previously reported (Kurtz *et al*, 1988; Rack *et al*, 2003; Montgomery *et al*, 2007).

One of the clear findings of this study is that the majority of second BCs was detected in asymptomatic women. These data suggest that ‘early detection’ of the second cancer may be occurring during routine follow-up of women with BC. A recent case–control study found mammography surveillance was associated with improved survival in older BC survivors (Lash *et al*, 2007). As we are considering survival from diagnosis of the second cancer, estimation of the effect of early detection (based on symptom status) is likely to be subject to lead-time and length biases. For this reason we have not included this variable in survival analysis for purposes of this study. However, we are currently collecting additional data on symptoms and mode of diagnosis of second cancers in our cohort to validly quantify the extent and potential impact of early detection in women with second cancer events.

The high proportion of asymptomatic second cancers is consistent with finding that overall second cancers were smaller than first cancers, although clearly evident for only CBC. As CBC is essentially a new cancer event, screening may play a larger role in its diagnosis. Ipsilateral breast relapses are predominantly recurrences of the initial tumour and so the observed differences in tumour size may reflect the differing biological nature of these two cancer events.

Histological types of tumours, for both the first and second cancer, also differed in distribution between IBR and CBC. This needs to be interpreted in the context of the long timeframe of the study (with possible variability in pathology reporting criteria and pathologists) and that some types were not specified. The interesting aspect of differences in the distribution of tumour histology is that it was largely determined (in second cancers) by a significantly higher proportion of DCIS in IBR than CBC, and a significantly higher proportion of invasive lobular cancer in CBC than IBR. Differences in distribution of histology of the first cancer were similarly influenced by a significantly higher proportion of DCIS in IBR than CBC.

Poorer disease-specific survival was associated with shorter times from the first to second cancer event, diagnosis of the second cancer in an earlier time period, second cancer tumour size ⩾2 cm, and positive nodes. The association between poorer prognosis and short time from the initial cancer to in-breast relapse is well known, but such an association has not previously been clearly demonstrated for CBC (Ciatto *et al*, 2004). The association between earlier epoch of diagnosis and poorer survival has been reported by others in analyses of population data in both primary early and metastatic BC (Chen *et al*, 2007; Ernst *et al*, 2007; Dabakuyo *et al*, 2008), so we presume this is mainly indicative of therapy effect. It is also possible that some of this effect relates to early detection.

Although all the prognostic variables we have described are relevant to clinicians providing care to women with a second BC, based on the strongest predictor variables for survival in our model, pT and pN categories are likely to be more clinically relevant in prognostication, although the occurrence of a second cancer soon after the first may be a warning sign of a more aggressive tumour and poorer outcome. The most powerful predictive features of the first BC, in terms of predicting second cancers with poor prognostic features (second cancer tumour size ⩾2 cm or positive nodes) were larger tumour size and positive nodes. A larger first BC (⩾2 cm) was a significant predictor of node metastases on diagnosis of the second cancer.

## Figures and Tables

**Table 1:** Second breast cancers in Tuscan women: key characteristics and outcomes

**Variable**	**Category/quantity**	**IBR (*N*=455)**	**CBC (*N*=589)**	**All second cancers (*N*=1044)**	***P*-value^a^**
Follow-up time from first cancer^b^	Median (IQR), years	13.3 (9.0–17.1)	14.5 (9.1–18.7)	13.7 (9.0–18.1)	NA
Time from second cancer to breast cancer death	Median (IQR), years	3.1 (1.9–5.3)	3.8 (1.9–6.2)	3.3 (1.9–5.7)	NA
Breast cancer deaths		78	103	181	NA
Deaths from other causes		14	48	62	NA
					
*Features of second cancer*					
Time from first to second cancer (DFI)	Median (IQR), months	57.4 (30.5–107.6)	68.0 (36.0–122.1)	63.4 (33.2–117.0)	0.029
Age at second cancer	Median (IQR), years	59 (50–69)	61 (53–71)	60 (51–70)	0.082
Presentation of second cancer	Asymptomatic (%)	314 (69)	385 (65)	699 (67)	0.214
	Symptomatic (%)	141 (31)	204 (35)	345 (33)	
pT category of second cancer	pTis (%)	70 (15)	55 (9)	125 (12)	0.015
	pT1a–c (%)	283 (62)	397 (67)	680 (65)	
	pT2+ (%)	71 (16)	105 (18)	176 (17)	
	pTx (%)	31 (7)	32 (5)	63 (6)	
Node status of second cancer	Negative (%)	NA	330 (56)	NA	NA
	Positive (%)	NA	116 (20)	NA	
	Not examined (%)	NA	143 (24)	NA	
Histology of second cancer	DCIS (%)	72 (16)	55 (9)	127 (12)	<0.0001
	Invasive ductal (%)	238 (52)	346 (59)	584 (56)	
	Invasive lobular (%)	55 (12)	115 (20)	170 (16)	
	Other special types (invasive)^c^ (%)	22 (5)	43 (7)	65 (6)	
	Other breast cancers (%)	68 (15)	30 (5)	98 (9)	
Surgery for second cancer	Mastectomy (%)	248 (55)	233 (40)	481 (46)	NA
	WLE (%)	183 (40)	342 (58)	525 (50)	
	Data missing (%)	24 (5)	14 (2)	38 (4)	
					
*Features of first cancer*					
Age at first cancer	Median (IQR), years	51 (43–63)	53 (45–62)	53 (44–62)	0.035
pT category of first cancer	pTis (%)	85 (19)	37 (6)	122 (12)	<0.0001
	pT1a–c (%)	257 (56)	271 (46)	528 (51)	
	pT2+ (%)	85 (19)	227 (39)	312 (30)	
	pTx (%)	28 (6)	54 (9)	82 (8)	
Node status of first cancer	Negative (%)	283 (62)	394 (67)	677 (65)	<0.0001
	Positive (%)	74 (16)	156 (26)	230 (22)	
	Not examined (%)	98 (22)	39 (7)	137 (13)	
Histology of first cancer	DCIS (%)	78 (17)	34 (6)	112 (11)	<0.0001
	Invasive ductal (%)	273 (60)	402 (68)	675 (65)	
	Invasive lobular (%)	58 (13)	98 (17)	156 (15)	
	Other special types (invasive)^c^ (%)	28 (6)	36 (6)	64 (6)	
	Other breast cancers (%)	18 (4)	19 (3)	37 (4)	

DCIS=ductal carcinoma *in situ*; IQR=interquartile range; NA=not applicable.

a Comparison of IBR with CBC where applicable.

b Based on 1016 subjects (from 1044 subjects) who had at least one episode of follow-up (from second cancer diagnosis) and verified outcomes through linkage with regional cancer or mortality registry.

c Includes cases where histological type of breast malignancy was not specified and those with missing data on type of cancer histology.

**Table 2:** Pathological tumour size (pT) category of the second breast cancer according to size of the first cancer in Tuscan women

	**Ipsilateral breast relapse (% from 455)**				**Contralateral breast cancer (% from 589)**				**Subjects with second cancer (% from 1044)**				
**First cancer**	**pTis**	**pT1a-c**	**pT2+**	**pTx**	**pTis**	**pT1a-c**	**pT2+**	**pTx**	**pTis**	**pT1a-c**	**pT2+**	**pTx**	**Total (%)**
pTis	32	44	7	2	6	21	10	0	38	65	17	2	122 (12)
pT1a–c	33	168	43	13	27	199	34	11	60	367	77	24	528 (51)
pT2+	5	52	17	11	15	141	55	16	20	193	72	27	312 (30)
pTx	0	19	4	5	7	36	6	5	7	55	10	10	82 (8)
Total (%)	70 (15)	283 (62)	71 (16)	31 (7)	55 (9)	397 (67)	105 (18)	32 (5)	125 (12)	680 (65)	176 (17)	63 (6)	1044 (100)

**Table 3:** Disease-specific survival analysis following diagnosis of a second breast cancer: multivariate Cox proportional hazards model

**Variable**	**Hazard ratio**	**95% confidence interval**	***P*-value**	**Number of cases**	**LR test statistic**	**Global *P*-value**
*Age at second cancer (years)*						
<50	1.00			213		
50–69	0.80	0.56–1.14	0.220	528	3.99	0.136
>69	1.15	0.77–1.71	0.505	267		
						
*Time from first to second cancer*						
⩾5 years	1.00			529	5.11	0.024
<5 years	1.45	1.05–2.00	0.026	479		
						
*Time period of second cancer*						
1998–2005	1.00			549	19.36	<0.0001
1980–1997	2.20	1.53–3.16	<0.0001	459		
						
*pT second cancer*						
T1	1.00			661		
T*is*	0.29	0.14–0.64	0.002	121	39.53	<0.0001
T2–4	2.26	1.57–3.25	<0.0001	168		
Tx	1.73	1.06–2.83	0.028	58		
						
*pN second cancer*						
pN0	1.00			376		
pN+	3.43	2.20–5.36	<0.0001	134	38.70	<0.0001
pNx (unknown)	3.35	2.00–5.62	<0.0001	138		
pNr (previously resected)	1.65	0.85–3.20	0.143	360		
						
*Second cancer*						
CBC	1.00			573	0.63	0.427
IBR	1.29	0.70–2.40	0.413	435		

**Table 4:** Multivariate logistic regression for assessing features of the first breast cancer associated with large or node-positive second breast cancers

	**Predictive of second cancer with tumour size ⩾ 2 cm**				**Predictive of second cancer with positive nodes**			
**First cancer variable**	**Odds ratio (95% CI)**	***P*-value**	**LR test statistic**	**Global *P*-value**	**Odds ratio (95% CI)**	***P*-value**	**LR test statistic**	**Global *P*-value**
*Age at first cancer (years)*								
<50	1.00				1.00			
50–69	0.96 (0.67–1.38)	0.837	1.85	0.396	0.76 (0.52–1.12)	0.171	3.71	0.157
>69	1.37 (0.82–2.28)	0.234			0.57 (0.29–1.11)	0.097		
								
*pT of first cancer*								
T1	1.00				1.00			
T*is*	1.67 (0.44–6.38)	0.452	7.46	0.059	2.18 (0.57–8.35)	0.254	17.36	0.0006
T2–4	1.54 (1.06–2.23)	0.023			2.44 (1.59–3.75)	<0.0001		
Tx	0.75 (0.37–1.55)	0.439			1.40 (0.68–2.90)	0.360		
								
*pN of first cancer*								
pN0	1.00				1.00			
pN+	1.51 (1.02–2.22)	0.039	4.64	0.098	1.68 (1.10–2.59)	0.017	6.92	0.031
pNx	1.38 (0.73–2.64)	0.324			1.73 (0.86–3.46)	0.122		
								
*Histology of first cancer*								
Invasive ductal	1.00				1.00			
DCIS	0.44 (0.11–1.73)	0.241	2.27	0.518	0.63 (0.16–2.44)	0.506	4.35	0.227
Invasive, special types	0.72 (0.31–1.63)	0.427			2.10 (1.02–4.32)	0.043		
Invasive (other)^a^	1.11 (0.73–1.68)	0.620			1.08 (0.68–1.73)	0.737		

a Cases where histological type of invasive breast malignancy was not specified and those with missing data on type of cancer histology.
